# Seasonal dynamics and potential interactions of haematophagous abomasal nematodes in two chamois populations in the Czech Republic

**DOI:** 10.1186/s12917-025-04992-6

**Published:** 2025-09-01

**Authors:** Jan Magdálek, Vojtěch Kasič, Jana Ilgová, Lucie Škorpíková, Jaroslav Vadlejch

**Affiliations:** 1 Department of Zoology and Fisheries, Faculty of Agrobiology, Food and Natural Resources, Czech University of Life Sciences Prague, Kamýcká 129, Prague Suchdol, 165 00 Czech Republic; 2 Department of Game Management and Wildlife Biology, Faculty of Forestry and Wood Sciences, Czech University of Life Sciences Prague, Kamýcká 129, Prague Suchdol, 165 00 Czech Republic; 3 Department of Botany and Zoology, Faculty of Science, Masaryk University, Kotlářská 2, Brno, 611 37 Czech Republic

**Keywords:** Chamois, *Haemonchus contortus*, *Ashworthius sidemi*, Real-time PCR, Prevalence, Epidemiology

## Abstract

**Background:**

Pathogenic blood-feeding nematodes, such as *Haemonchus contortus* and the invasive *Ashworthius sidemi*, infect a wide range of wild and domestic ruminants. While the spread of *A. sidemi* among European cervids has been studied, its presence in chamois (*Rupicapra rupicapra*) remains poorly documented. Conversely, *H. contortus* is known to infect chamois, but previous research has relied mainly on cross-sectional necropsy studies, offering only a limited view of infection dynamics. In this study, we used a longitudinal molecular approach to assess the seasonal occurrence and transmission patterns of *H. contortus* and *A. sidemi* in a chamois population from the northern Czech Republic. From January to December 2023, we collected faecal samples at monthly intervals from two localities. Multiplex real-time PCR was subsequently used for the detection and semi-quantification of DNA from both nematode species.

**Results:**

*Haemonchus contortus* DNA was detected in 43.3% of samples, with its presence recorded nearly year-round. Its prevalence and relative DNA quantity peaked in summer and remained high throughout autumn. *Ashworthius sidemi* was identified in chamois in the Czech Republic for the first time, possibly reflecting a recent spillover from red deer (*Cervus elaphus*). However, it was found in only 5% of samples, with its occurrence restricted to late winter and spring. The seasonal disappearance of *A. sidemi* coincided with the increase in *H. contortus* relative DNA quantity, which may indicate a possible negative interaction between these species occupying the same ecological niche.

**Conclusion:**

Our findings suggest a potentialy prolonged transmission window for *H. contortus*, which could lengthen further under future climate change scenarios. In contrast, *A. sidemi* appears to be an incidental parasite in chamois, and its long-term persistence in this atypical host without continued contact with cervids remains uncertain. These insights, which are rare for wild ruminants, contribute to a better understanding of parasite epidemiology and host-parasite interactions in free-living populations.

**Supplementary Information:**

The online version contains supplementary material available at 10.1186/s12917-025-04992-6.

## Background

The chamois *Rupicapra rupicapra* is distributed to higher altitudes and specific, rocky terrain types; however, the species exhibits remarkable adaptability, allowing it to thrive in a wide range of mountainous environments [1]. The chamois is considered a non-native species in the Czech Republic (CR) as the presence of two populations of chamois in the country has its origin in introductions that took place at the beginning of the 20th century to enrich the spectrum of the species for trophy hunting. These introductions were repeatedly carried from the alpine regions of Austria and Germany, as well as from zoos and game breeders in Austria and Switzerland [2]. Analysis of the mitochondrial control region shows that the current chamois populations in the CR, found in the northern part of the country (The Lusatian Mountains Protected Area (PLA) and Jeseníky Mountains, are most genetically related to northern chamois populations in the eastern Alps, specifically in the Ebensee and Mürzsteg regions [3]. As the mountain chamois is restricted to specific habitats and has low dispersal rates, gene flow is limited, resulting in genetically distinct populations. Introduced populations in Central Europe have shown lower genetic variability than their Alpine counterparts, and apparent consequences of founder effects and bottleneck events [4], which could potentially increase their susceptibility to pathogens.

Chamois host a variety of gastrointestinal parasites, with those infecting the abomasum being the most significant. While typically subclinical [5], these infections can negatively impact the host’s fitness and welfare. A negative correlation between the species richness of abomasal nematodes and both skeletal development and nutritional status of alpine chamois in Italy has been observed [6]. Among these parasites, *Haemonchus contortus* is considered the most important for chamois health [7, 8]. Although primarily associated with significant losses in small livestock farms worldwide [9], this blood-sucking nematode has low host specificity and can spread between grazing livestock and wild ruminants, including northern chamois [10]. This parasite was identified as the direct cause of death in only 1.4% of postmortem-examined chamois in the Slovenian Alps [11]. However, its broader impact should not be underestimated as subclinical infections may weaken individuals, making them more susceptible to other stressors. In the Italian Alps, *H. contortus* was reported as a predominant abomasal parasite and suggested as a predisposing factor contributing to a pneumonia-related mass die-off [12].

In addition, a close relative of the parasite *H. contortus* from the subfamily Haemonchinae, *Ashworthius sidemi*, has recently been identified in a hybrid of alpine (*R. rupicapra rupicapra*) and tatra chamois (*R. rupicapra tatrica*) from the Low Tatras (Slovakia) [13]. The original hosts of this nematode are thought to be the cervids of eastern Asia, but with the translocation of sika deer (*Cervus nippon*), it was inadvertently repeatedly introduced into central and eastern Europe and France, as reviewed by Rehbein et al. [14]. It has successfully transferred from its original host to various local ruminant species and, in Europe, currently occurs locally with a high prevalence [15]. In addition, with new hosts, there is likely to be further natural spread of the parasite into new territories [14, 16–18]. The parasite was first recorded in the former Czechoslovakia in the 1970 s at the Lány game reserve [19], and recent molecular monitoring indicates that it is now most common in red deer (*Cervus elaphus*) [20]. In that study, the *A. sidemi* infection was also confirmed in red deer in the locality of chamois population occurrence in the northern part of the CR. Despite the different feeding strategies of red deer and chamois [21], the diet composition observed in the other chamois locality, the Jeseníky Mountains, was almost identical for both species. Both diets were primarily composed of grasses, with smaller proportions of herbs and woody broad-leaved plants [22]. This overlap in ecological niches suggests the potential for transmission of the invasive *A. sidemi*.

Gastrointestinal nematode data from chamois have typically been collected through necropsy-based cross-sectional studies, e.g [7, 12, 13]. While these methods enable precise species identification and infection intensity estimates, they provide only a narrow insight into fluctuating infection status, as sampling is usually limited to the autumn hunting season.

Alternatively, coprological analysis offers a non-invasive way to monitor seasonal parasite output [23, 24]. However, this method does not allow species-level identification, as most strongylid parasite eggs are morphologically indistinguishable, limiting its ability to track the most pathogenic species. For these reasons, we adopted the real-time PCR method proposed by Reslová et al. [25], which enables reliable differentiation of the haematophagous nematodes *A. sidemi* and *H. contortus*, as well as semi-quantification directly from faecal samples.

The main goals of this study were to investigate the occurrence of the invasive species *A. sidemi* and the important pathogen *H. contortus* in a northern chamois population from the Lusatian Mountains PLA (CR) and to monitor seasonal variations in egg production of both parasites as well as explore potential interactions between them.

## Methods

### Study site and animals

The chamois population in the northern part of the Czech Republic inhabits three protected areas: the Czech Switzerland National Park, the Lusatian Mountains PLA, and the Elbe Sandstones PLA. To monitor the occurrence and seasonal variation in *A. sidemi* and *H. contortus* egg production, we selected two areas with the highest chamois presence: one in the Lusatian Mountains PLA and the other in the Elbe Sandstones PLA. These study sites were approximately seven km apart aerially, separated by fields, urban development, and a traffic road. Based on the Köppen–Geiger climate classification [26], the study area is characterized as a warm-summer humid continental climate, typical for Central Europe. This climate features clearly defined seasons, with warm but not excessively hot summers, long and cold winters, and generally lower levels of precipitation throughout the year [27]. The locality A, Studený Vrch, lies adjacent to the Studenec Hill (50°49′56″ N, 14°27′16″ E) with its peak at an altitude of 737 m. The terrain consists of volcanic stony fields, partly refugial post-glacial basiphilous beech mixed fir-beech forests (alliance *Fagion sylvaticae*) and meadows dominated by false oatgrass (*Arrhenatherum elatius*) grazed by chamois during the vegetation period. Based on the census carried out by the local forestry service, the hunting ground covering this site was inhabited by 39 individuals in 2023.

From January to April, supplementary feeding of concentrates was carried out, while from March onwards, the movement to meadows and grazing was allowed. From the end of January until approximately mid-March, a continuous snow cover was observed at this site, except for one week at the end of February. Feeding took place on common feeders with red deer, whose numbers were estimated at 78 individuals on the hunting ground. During the grazing season, deer and chamois used the same meadows, but both species grazed mostly in separate parts. Mixed groups were not observed during the daytime.

The second monitored site (locality B) was located in the Česká Kamenice hunting ground, specifically on Strážiště Hill (50°48′59″N, 14°22′19″E) at an altitude of 469 m. This area is characterised by generally lower elevation and is dominated by Man-made forests with high prevalence of *Picea* sp. and vegetation from the *Luzulo-Fagetum* association. The number of chamois at this site was estimated at 47 individuals by the local forestry service. Supplementary feeding was limited here to the winter season and occurred at feeding stations specially adjusted to be accessible only to chamois without disturbance by the red deer. Simultaneously, grazing was carried out all year round, with mixed groups of chamois and deer observed at this site. The total number of deer on this hunting ground was estimated at 126.

### Sample collection

Freshly excreted faecal samples were collected monthly from both sites. The animals were first observed with binoculars outside their flight distance to minimize stress. To reduce the risk of species misidentification, sampling was conducted only when no other ungulate species were present in the area. After the chamois had left the site, efforts were made to collect samples from ten different individuals without resampling the same animal. Only visibly fresh samples were collected as soon as possible after defecation, to minimize the risk of environmental contamination. In total, 240 faecal samples were gathered between January 2023 and December 2023. The health status, sex, and age of the sampled animals could not be determined from the observation distance. The samples were sealed separately in zip-lock bags, transported to the laboratory, where 5 g of each sample were weighed, resealed, and stored at −20 °C for later molecular analyses.

### Molecular analysis

Total DNA was extracted from faecal samples using the Quick-DNA Fecal/Soil Microbe MiniPrep Kit (Zymo Research, Irvine, CA, USA), following a protocol adapted from [25]. Frozen samples were thawed at room temperature and homogenized manually. From each sample, 1 g of feces was suspended in 800 µL BashingBead Buffer and 3,200 µL PBS, then mixed thoroughly. A 1,200 µL aliquot was transferred to a ZR BashingBead Lysis Tube and lysed using a Retsch MM200 mixer mill (1,800 rpm, 10 min). Each extraction batch (~ 30 samples) included a negative isolation control. DNA purification was completed according to the manufacturer’s instructions, and final eluates (50 µL) were stored at −20 °C until further analysis.

Specific primers and TaqMan probes targeting the *ITS1* region of *A. sidemi* and the *ITS2* region of *Haemonchus* spp. were used in a triplex real-time PCR assay, which also included a synthetic internal amplification control (IAC) to detect potential PCR inhibition. Cross-reactivity with non-target species has been previously tested and validated in detail, as described in [20, 25]. The reaction composition, primer/probe concentrations, and thermal cycling parameters were adopted from previously published protocols [25]. Briefly, each 20 µL reaction contained Luna Universal Probe qPCR Master Mix, 250 nM of each primer, 100 nM of FAM probe, 100 nM Cy5 probe, 200 nM of HEX probe, 0.4 U of Antarctic Thermolabile UDG (New England Biolabs, Ipswich, MA, United States), 1 × 10^5^ copies of IAC plasmid, 5 µL of template DNA, and PCR-grade water. Amplification was performed in duplicate on a CFX96 Real-Time PCR Detection System (Bio-Rad Laboratories, Hercules, CA, USA) under following cycling conditions: 37 °C for 10 min (carryover prevention), 95 °C for 2 min, followed by 40 cycles of 95 °C for 15 s and 57 °C for 45 s, with final cooling at 40 °C for 30 s. Data were analyzed using CFX Manager 3.0 software (Bio-Rad Laboratories).

To enable detection and semi-quantification of both parasites, the relative quantity of DNA (expressed as plasmid copy number equivalents) was determined for each target using Ct values adjusted for inter-target differences with an empirically derived correction factor [25]. These values were subsequently interpolated against a standard curve generated from tenfold serial dilutions (ranging from 5 × 10⁷ to 5 × 10³ copies) of a plasmid construct containing the *ITS2* sequence of *H. contortus*.

### Data analysis

For each sample, the presence or absence of *A. sidemi* and *H. contortus* (coded as 1 and 0) and the relative amounts of target DNA in positive samples were recorded and used as dependent variables. All data analyses were performed in R 4.3.2. Due to a higher-than-expected number of zero values in the dataset (zero inflation), the analysis was conducted in two steps. First, the probability of presence or absence was analyzed using a generalized linear mixed model (GLMM) with a binomial distribution and a logit link function. Second, to account for overdispersion, a GLMM with a negative binomial distribution was used to examine the variability in the relative quantity of parasite DNA in positive samples. Locality (site), season, and their interaction were included as fixed effects to assess whether the effect of site on parasite presence varied by season. Additionally, a random effect (1 | month: locality) was incorporated to account for month-to-month variability within each site.

The models were implemented using the *glmmTMB* package, and data visualisation was performed in the ggplot2 package. The explanatory variable *season* was defined to categorize the study period into biologically meaningful timeframes. Winter (January–March) was characterized at Site A by continuous snow coverage and intensive additional feeding in shared feeders with deer, while at Site B, additional feeding was limited and restricted to the chamois-only feeder, with continuous grazing throughout. Spring (April–June) marked the transition to grazing at Site A and, at both sites, coincided with the peak phase of female gestation. The summer period (July–September) was defined by the presence of young individuals grazing and undergoing weaning at its peak, with mixed herds of chamois and red deer observed at Site B. Autumn (October–December) was characterized by the rutting season for males at both sites. Model diagnostics were performed using the DHARMa package. For samples that tested positive for both parasites simultaneously, a Spearman’s rank correlation test was conducted to assess potential interactions between the two species. Additionally, a Mann-Whitney U test was used to compare the relative quantity of *A. sidemi* DNA between samples with *A. sidemi* single infections and those with co-infections with *H. contortus.*

## Results

**Fig. 1:**
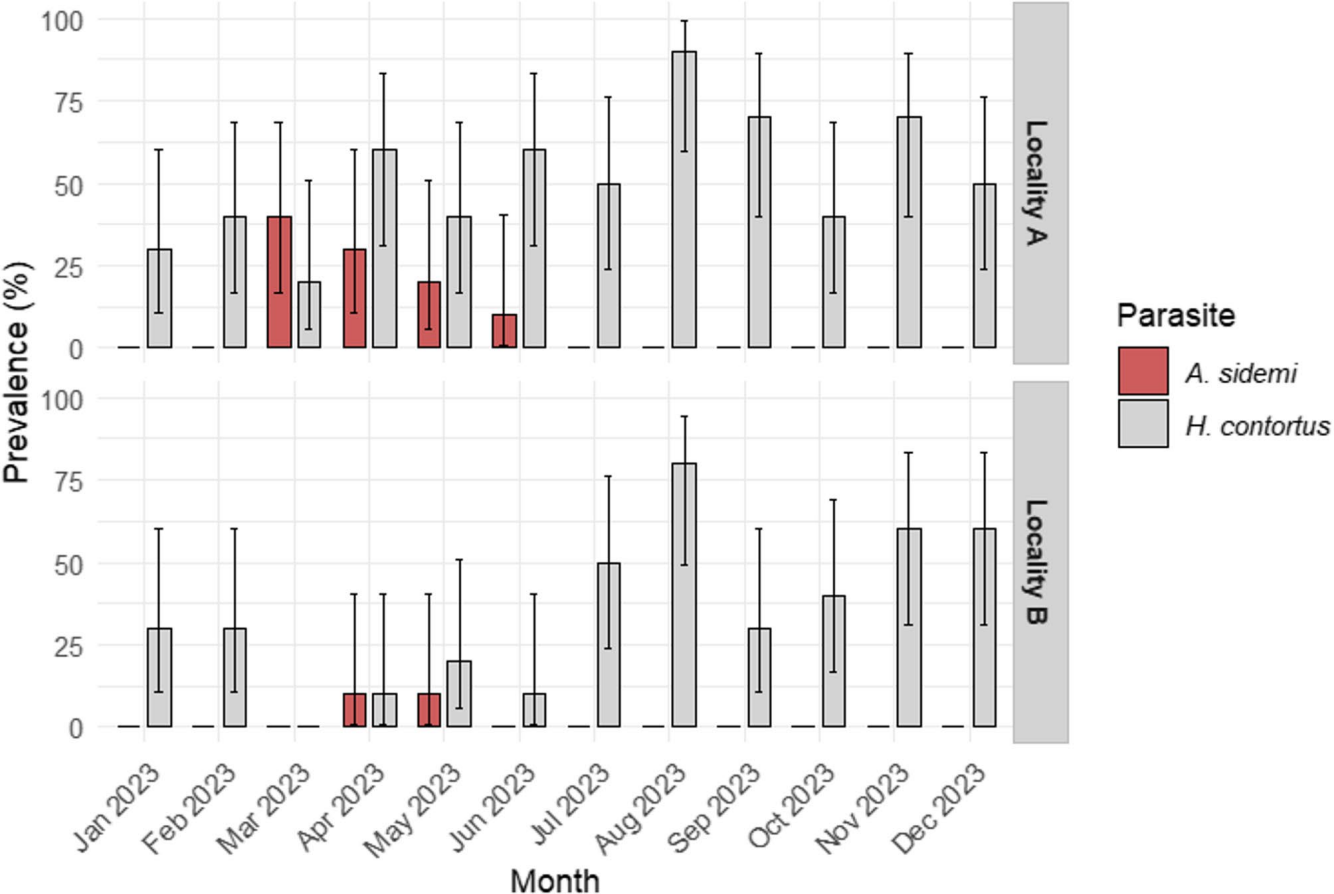
Percentage of positive samples with 95% confidence intervals for *A. sidemi* and *H. contortus* in the monitored months at locality A (Studený Vrch) and B (Česká Kamenice)

The presence of DNA from both parasite species (*A. sidemi* and *H. contortus*), was detected in the faeces of chamois at both monitored sites. *H. contortus* DNA was identified in 116 out of 240 samples, with a prevalence of 43.3% (95% CI = 36–49), while *A. sidemi* was found in only 12 samples (5%; 95% CI = 2–8). At Site A (Studený vrch), 62 out of 120 samples (51.7%; 95% CI = 42–60) tested positive for *H. contortus*, and 10 out of 120 (8.33%; 95% CI = 4–14) for *A. sidemi*. At the Česká Kamenice hunting ground (Site B), *A. sidemi* was detected in only two out of 120 samples (1.67%; 95% CI = 0.2–5.8), while *H. contortus* was found in 42 out of 120 samples (35%; 95% CI = 26–44). The monthly prevalence is presented in Fig. 1.

At Site A, *A. sidemi* was detected continuously from March to June, with the highest proportion of positive samples (4/10) recorded in March. The frequency of positive samples then gradually declined, with three cases confirmed in April, two in May, and one in June. No positive samples were recorded during the summer and autumn periods. At Site B, *A. sidemi* exhibited a similar seasonal pattern but at a lower frequency. During spring, it was detected only in April (1/10) and May (1/10), after which it was not observed. Due to the low prevalence of *A. sidemi*, the statistical model described in the Data Analysis section could not be applied to this species. As a result, neither the probability of presence nor the relative DNA quantity in positive samples was analyzed. However, at Site A—and to a limited extent at Site B—a rising trend in the relative amount of parasite DNA was observed in spring samples.

*H. contortus* DNA was detected in samples from all months at both sites, except for March at Site B, with frequencies ranging from 10 to 90% (Fig. 1). The highest values were recorded at both sites in August. As shown in Table 1, at Site B, the probability of *H. contortus* occurrence was significantly lower in the spring season (*p* < 0.05). Additionally, results indicated a lower prevalence during winter months at both sites, although this effect was not statistically significant (*p* = 0.069).

**Table 1:** Results of the generalized linear mixed model (GLMM) for probability of *Haemonchus contortus* DNA presence

Response variable	Factor	Estimate ± SD	Z-value	*p*-value
Presence/absence of *H. contortus*	(Intercept)	0.134 ± 0.366	0.365	0.715
	Winter	−0.980 ± 0.541	−1.813	0.069
	Spring*Locality B	−2.005 ± 0.830	−2.414	0.016
Random effect	Variance	SD		
Month: locality	2.264e-09	4.759e-05		

**Fig. 2:**
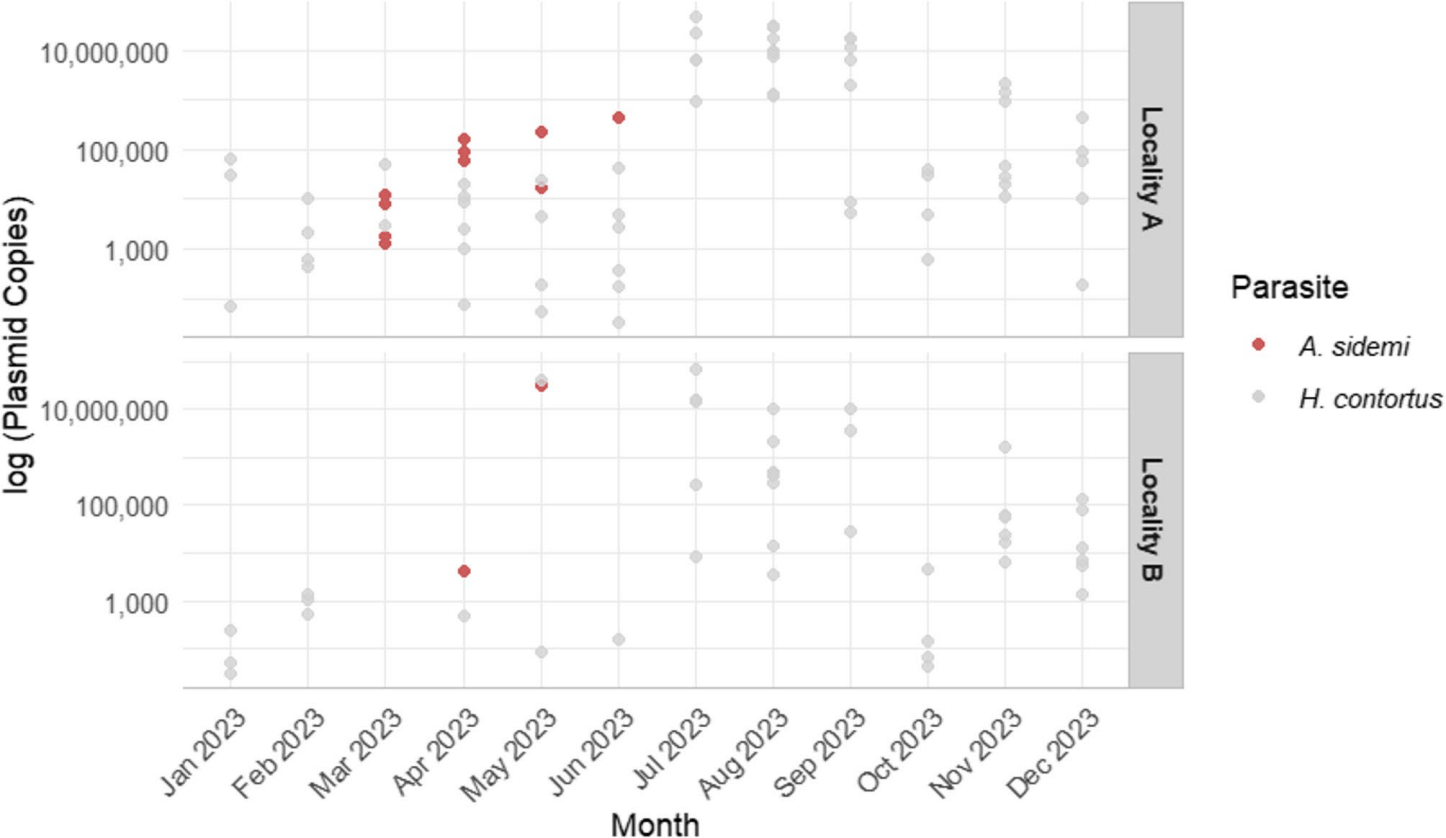
Seasonal variation in the amount of target DNA for *A. sidemi* (red points) and *H. contortus* (grey points) in positive samples collected at locality A (Studený Vrch) and B (Česká Kamenice) from January 2023 to January 2024. DNA levels are expressed as the plasmid standard copy number on a log-scaled y-axis

The relative quantity of *H. contortus* DNA varied seasonally, as shown in Fig. 2. The highest values were detected in summer (*p* < 0.001), followed by a decline in autumn. In contrast, the lowest DNA levels were recorded in winter (*p* = 0.005) and spring (*p* < 0.001). The model results, summarized in Table 2, revealed an interaction between spring and Site B, with significantly higher levels of *H. contortus* DNA in positive samples from Site B during the spring season (*p* < 0.001). However, this result was likely influenced by an extreme value and the low prevalence at this site during that period. Overall, no significant difference was found in the relative quantity of *H. contortus* DNA between the two sites (*p* = 0.231). Full model outputs and results of DHARMa residual diagnostics are available in Additional File 1. In total, eight samples tested positive for the DNA of both parasites simultaneously, while four samples were positive only for *A. sidemi*. A significant positive correlation was found between the relative quantity of *A. sidemi* and *H. contortus* DNA in co-infected samples (Spearman’s rank correlation: ρ = 0.976, *p* = 0.0004). However, the comparison of *A. sidemi* DNA levels between single infections and co-infections showed no statistically significant difference (Mann-Whitney U test: W = 7, *p* = 0.153).

**Table 2:** Results of the generalized linear mixed model (GLMM) for relative amount of *Haemonchus contortus* DNA in positive samples

Response variable	Factor	Estimate ± SD	Z-value	*p*-value
Level of *H. contortus* DNA	(Intercept)	12.42 ± 0.680	18.256	< 0.001
	Spring	−3.408 ± 0.905	−3.766	< 0.001
	Summer	3.931 ± 0.868	4.528	< 0.001
	Winter	−2.617 ± 0.947	−2.762	0.006
	Locality B	−0.994 ± 0.831	−1.195	0.231
	Spring*Locality B	8.083 ± 1.417	5.705	< 0.001
Random effect	Variance	SD		
Month: Locality	0.257	0.507		

## Discussion

In this study, we investigated seasonal changes in contamination by eggs of two haematophagous nematodes, *Haemonchus contortus* and an invasive species *A. sidemi*, in the faeces of chamois at two sites in the Northern part of the Czech Republic.

### *Ashworthius sidemi*

The occurrence of *A. sidemi* in the studied chamois feacal samples was rather episodic as the DNA of this parasite was detected at Site A continuously from late winter through spring 2023, while at Site B, it was found only in April and May. To our knowledge, this is the first confirmed occurrence of *A. sidemi* in chamois in the CR. The only relevant previous record of *A. sidemi* infection in chamois comes from the Low Tatras (Slovakia), where it was detected in a single crossbred Tatra (*R. r. tatrica*) and Northern chamois (*R. r. rupicapra*) based on a digestive tract necropsy [13], while high prevalences were recorded in sympatric cervids. *A. sidemi* is generally considered a species typically associated with hosts from the subfamily Cervinae [15]. Although it has been confirmed as a generalist capable of infecting both free-living and domestic bovids [28], its occurrence in free-living bovids appears to be limited. The main exception appears to be the European bison, in which the parasite is well established, as evidenced by records from Poland and the CR [29–31].

The origin of chamois in the northern part of the CR is complex, as the founding individuals were imported from various zoos and private breeding facilities in the early 20th century. Since these animals were unlikely to have been in contact with sika deer, the primary host of *A. sidemi*, and this parasite has not been recorded in chamois in their countries of origin, such as Austria [32], or only appeared later due to its spread, as in Bavaria [14], it is unlikely that *A. sidemi* was introduced with the original chamois population. Additionally, molecular analysis recently confirmed the presence of *A. sidemi* eggs in red deer faeces at a site adjacent to the North Bohemian chamois population monitored in this study [20].

These findings suggest a possible transmission of *Ashworthius sidemi* to chamois from sympatric red deer, in which this parasite is actively spreading in the Czech Republic. Although direct evidence of cross-species transmission is lacking, deer populations are considered important drivers of *A. sidemi* expansion into new areas across Europe [14, 16]. This interpretation is further supported by our detection of several nematodes, *Spiculopteragia spiculoptera* and *Ostertagia leptospicularis*, species typically associated with cervids [15, 32], in the abomasum of a single chamois culled at Site A during a fee hunting event in autumn 2022 (unpublished data).

When we compare both monitored sites, the slightly higher percentage of positivity and earlier onset of *A. sidemi* appear at Site A. This may be attributed to closer contact with red deer, as supplementary feeding was provided at shared feeders with red deer until April. In contrast, at Site B, chamois primarily relied on grazing, and only a feeding station was designed to restrict deer access, with supplementary feeding being less intensive.

Additionally, increased stress levels could have led to higher susceptibility to the parasite. For instance, in Apennine chamois (*Rupicapra pyrenaica ornata*), faecal cortisol metabolite levels were found to rise during periods of habitat overlap with red deer [33].

Overall, the occurrence of *A. sidemi* in chamois was limited to the spring and winter seasons, contrasting with a previous longitudinal study on farmed fallow deer (*Dama dama*), where *A. sidemi* DNA was detected in pastures nearly year-round, except in the months following anthelmintic treatments [34]. However, the absence of *A. sidemi* DNA for most of the year does not necessarily indicate the parasite’s absence in the host’s abomasum. Egg shedding may not directly reflect parasite load, as it can be influenced by reduced production or the presence of sexually immature stages [24]. *A. sidemi* is known to persist within the host for extended periods in its juvenile or larval stages [35, 36]. Consistent with this, our dissection of two chamois culled in autumn 2023 revealed dozens of juvenile *A. sidemi* stages (unpublished data), suggesting its ongoing persistence despite the lack of detectable DNA in environmental samples.

### *Haemonchus contortus*

*Haemonchus contortus* was present in the samples of chamois from almost all monitored months. This parasite is globally recognized as a significant pathogen affecting small domestic ruminants, particularly sheep and goats. To a lesser extent, it has also been recorded in cattle. The presence of this parasite in wild ruminants, such as chamois, is typically associated with shared grazing areas in proximity to domestic ruminants [12, 13] The low host specificity of *H. contortus*, combined with its ability to adapt to various ecological conditions, facilitates its circulation between domestic animals and mountain ungulates [10, 37]. At the time of our study, shared grazing with domestic ruminants was not recorded; however, as shown by a recent study of *H. contortus* transmission between domestic ruminants and roe deer (*Capreolus capreolus*) in France the presence of this in wild ruminants is probably more closely related to long-term ecological co-occurrence rather than merely the actual intensity of contact with livestock [38].

The seasonal dynamics of *Haemonchus contortus* in ruminants are influenced by various environmental factors, particularly temperature and moisture, which significantly affect the life cycle and transmission [39, 40].

To survive cold and dry periods, *H. contortus* larvae may enter a state of arrested development (hypobiosis), remaining in the abomasal tissue after being ingested by the host [41, 42]. They can then reactivate and continue their development when environmental conditions become favorable. During this period, detecting eggs in faeces is typically challenging. Although the relative quantity of *H. contortus* DNA detected in our study during winter was significantly lower than in the reference (autumn) season, the number of positive samples indicated ongoing presence of reproducing stages. However, the likelihood of transmission during winter is limited as the low temperatures typical for this period rapidly reduce the ability to hatch and subsequent larval survival and dispersion [43, 44]. The meteorological station in Česká Kamenice, located between the two study sites, recorded nearly 40 days with minimum temperatures below 0 °C between January and March 2023. While at Site A, a continuous snow cover was recorded during this time, at Site B, the developmental stages were exposed to frost most of the time. Snow coverage can act as an insulating layer that can moderate soil temperatures and influence its moisture levels [45], which can explain the significant decline in positive samples during spring and the absence of the parasite at Site B in March.

The highest prevalence and relative quantity of the parasite was found in the summer period, with a peak in August at both sites monitored. This was followed by a decline in autumn; however, the probability of parasite occurrence in autumn remained comparable to spring, while relative quantity was significantly higher than in spring samples. A similar pattern was observed in the United Kingdom, where historical data analysis showed that the number of ovine haemonchosis diagnoses peaked in late summer and persisted into early autumn [46]. Similarly, Citterio et al. [12] observed the dominance of *H. contortus* during the autumn hunting season in a five-year study of abomasal nematode communities in chamois in Italy. In our study, the parasite was detected in samples until January 2024. This timing of peak egg production in late summer and early autumn may enable the parasite to infect susceptible weaned young. If conditions remain favorable for hatching and larval development, these hosts may become infected and start shedding eggs within a few weeks. This could explain the slight increase in the relative amount of *H. contortus* DNA observed in our study in November. However, other host-related factors must also be considered. For instance, studies conducted in the western Italian Alps on chamois [47, 48] reported male-biased lungworm larval counts in autumn, coinciding with the rutting season, with territorial males exhibiting higher lungworm burdens. Due to the way our data were obtained, it was not possible in this study to assess host factors affecting egg production, such as age groups and sex of the sample studied.

Studies based on historical data and predictive models have shown that parasite transmission periods in temperate zones tend to increase with climate change [40, 46, 49]. While our observations are consistent with the expected extension of transmission periods, their interpretation is limited by the use of data from a single meteorological station, which may not capture local variability. Minimum daily temperatures measured near the study sites remained above 0 °C for most of November, suggesting potentially favourable conditions for parasite development. However, to accurate assesse the infectivity and transmission risk of parasite, it would be necessary to monitor the microclimate of the vegetation at the grazing sites.

### Possible interaction

A comparison of seasonal dynamics of *A. sidemi* and *H. contortus* raises the question of potential interactions between these species. A meta-analysis of gastrointestinal nematode co-infections in sheep suggests that *H. contortus* generally exerts an antagonistic effect on co-infecting species [50]. However, DNA analysis of spring samples from co-infected chamois showed no direct negative interaction.

Instead, *A. sidemi* and *H. contortus* exhibited a positive correlation in quantity, with no significant differences between *A. sidemi*-only infections and co-infections with *H. contortus*. Interestingly, the timing of *A. sidemi* disappearance from the samples coincided with a summer rapid increase in *H. contortus* levels at both sites. This was particularly surprising at site B, where the formation of mixed herds of chamois and red deer, a potential source of *A. sidemi* transmission, was recorded during this period. *H. contortus* is well known for its high biotic potential, allowing it to respond rapidly to even slight changes in external conditions [51]. In contrast, *A. sidemi* exhibited only a slight increase in pasture contamination during the summer, as reported in a previous study on its seasonal dynamics in fallow deer [34]. In the present study, the observed quantity of *H. contortus* DNA increased sharply during summer, likely due to rising temperatures that enhanced larval survival and development to infective stages. Subsequent large-scale infection by *H. contortus* may have triggered an immune response in the host, potentially reducing larvae infectivity and egg production of the *A. sidemi*. Such negative interactions of *H. contortus* on gastrointestinal nematodes have been previously observed, for example, on *T. circumcincta* [52] or *Nematodirus battus* [53]. However, these studies were conducted in laboratory settings using high doses of infective larvae, whereas our field study design allowed only relative quantification of potential infection. A more detailed assessment of the interaction between these two parasites will require further experimental research.

## Conclusions

This study provides insights into seasonal dynamics of egg output for two important gastrointestinal nematodes, *H. contortus* and *A. sidemi*, in chamois in the northern CR. The findings show distinct seasonal patterns for both parasites, with *H. contortus* showing a clear seasonal variation, peaking during the summer months. The year-round detection of parasite DNA suggests ongoing infection cycles and indicates the possibility of an extended transmission period in a temperate environment. However, further research, including microclimate monitoring and larval viability assessments, is needed to confirm actual transmission dynamics. In contrast, *A. sidemi* displayed a more episodic occurrence, with DNA primarily detected during the winter and spring months. This limited presence may reflect the lower susceptibility of the atypical host or the parasite’s persistence in non-mating forms, such as juvenile or larval stages, which do not contribute to egg shedding. Our study represents the first confirmed record of *A. sidemi* in chamois in the CR, and indirect evidence suggests that the parasite may have been transmitted from sympatric deer populations. Despite the absence of clear evidence of antagonistic interaction between *H. contortus* and *A. sidemi*, the observed patterns imply that shifts in parasite loads, such as the summer increase of *H. contortus*, may influence the dynamics of co-infection. This study contributes data on the ecology of gastrointestinal nematodes in wild ruminants, which can aid in the development of integrated management strategies to mitigate the impact of parasitic infections on both wild and domestic ruminant populations.

## Supplementary Information

Supplementary Material 1.

## Data Availability

The datasets used and analysed during the current study are available from the corresponding author on reasonable request.

## References

[1] Corlatti L, Iacolina L, Safner T, Apollonio M, Buzan E, Ferretti F, et al. Past, present and future of chamois science. Wildl Biol. 2022. 10.1002/wlb3.01025.

[2] Briedermann L, Still V. Die Gemse des elbsandsteingebietes. Rupicapra r. rupicapra. Die Neue Brehm-Bücherei. Wittenberg Lutherstadt: A. Ziemsen; 1976.

[3] Martínková N, Zemanová B, Kranz A, Giménez MD, Hájková P. Chamois introductions to central Europe and new Zealand. Folia Zool. 2012;61:239–45.

[4] Crestanello B, Pecchioli E, Vernesi C, Mona S, Martínková N, Janiga M, et al. The genetic impact of translocations and habitat fragmentation in chamois (*Rupicapra*) spp. J Hered. 2009;100:691–708. 19617524 10.1093/jhered/esp053

[5] Gunn A, Irvine R. Subclinical parasitism and ruminant foraging strategies - a review. Wildl Soc Bull. 2003;31:117–26.

[6] Zaffaroni E, Citterio C, Sala M, Lauzi S. Impact of abomasal nematodes on roe deer and chamois body condition in an alpine environment. Parassitologia. 1997;39:313–7. 9802085

[7] Zaffaroni E, Manfredi MT, Citterio C, Sala M, Piccolo G, Lanfranchi P. Host specificity of abomasal nematodes in free ranging alpine ruminants. Vet Parasitol. 2000;90:221–30. 10842002 10.1016/s0304-4017(00)00240-5

[8] Corlatti L, Herrero J, Ferretti F, Anderwald P, García-Gonzáles R, Hammer SE, et al. Northern chamois *Rupicapra Rupicapra* (Linnaeus, 1758) and Southern chamois *Rupicapra pyrenaica* bonaparte, 1845. In: Hackländer K, Zachos FE, editors. Handbook of the mammals of Europe - Terrestrial cetartiodactyla. Heidelberg: Springer Nature; 2022. p.325 – 57.

[9] Besier RB, Kahn LP, Sargison ND, Van Wyk JA. The pathophysiology, ecology and epidemiology of *Haemonchus contortus* infection in small ruminants. Adv Parasitol. 2016;93:95–143. 27238004 10.1016/bs.apar.2016.02.022

[10] Cerutti MC, Citterio CV, Bazzocchi C, Epis S, D’Amelio S, Ferrari N, et al. Genetic variability of *Haemonchus contortus* (Nematoda: Trichostrongyloidea) in alpine ruminant host species. J Helminthol. 2010;84:276–83. 19889245 10.1017/S0022149X09990587

[11] Vengušt G, Kuhar U, Jerina K, Švara T, Gombač M, Bandelj P, et al. Passive disease surveillance of alpine chamois *(Rupicapra r. rupicapra*) in Slovenia between 2000 and 2020. Animals. 2022. 10.3390/ani12091119. 35565546 10.3390/ani12091119 PMC9100901

[12] Citterio CV, Caslini C, Milani F, Sala M, Ferrari N, Lanfranchi P. Abomasal nematode community in an alpine chamois (*Rupicapra r. rupicapra*) population before and after a die-off. J Parasitol. 2006;92:918–27. 17152929 10.1645/GE-3551.1

[13] Nosal P, Kowal J, Wyrobisz-Papiewska A, Chovancová G. Ashworthius *sidemi* Schulz, 1933 (Trichostrongylidae: Haemonchinae) in mountain ecosystems – a potential risk for the Tatra chamois *Rupicapra rupicapra tatrica* (Blahout, 1971/1972). Int J Parasitol Parasites Wildl. 2021;14:117–20. 33598401 10.1016/j.ijppaw.2021.01.010 PMC7868822

[14] Rehbein S, Velling M, Visser M, Heurich M, Hamel D. Successful establishment of *Ashworthius sidemi* in red deer (*Cervus elaphus*) in germany, with a summary of worldwide *A. sidemi* records. Eur J Wildl Res. 2025;71: 2.

[15] Brown TL, Morgan ER. Helminth prevalence in European deer with a focus on abomasal nematodes and the influence of livestock pasture contact: a meta-analysis. Pathogens. 2024;13: 378. 38787230 10.3390/pathogens13050378 PMC11123710

[16] Demiaszkiewicz AW, Merta D, Kobielski J, Filip KJ, Pyziel AM. Expansion of *Ashworthius sidemi* in red deer and roe deer from the lower Silesian wilderness and its impact on infection with other gastrointestinal nematodes. Acta Parasitol. 2017;62:853–7. 29035860 10.1515/ap-2017-0103

[17] Kuznetsov DN, Romashova NB, Romashov BV. Gastrointestinal nematodes of European roe deer (*Capreolus capreolus*) in Russia. Russ J Theriol. 2020;19:85–93.

[18] Kuznetsov D. The first detection of abomasal nematode *Ashworthius sidemi* in fallow deer (*Dama dama*) in Russia. Acta Parasitol. 2022;67:560–3. 34263441 10.1007/s11686-021-00452-x

[19] Kotrlá B, Kotrlý A. The first finding of the nematode *Ashworthius sidemi* schulz,1933 in Sika Nippon from Czechoslovakia. Folia Parasitol. 1973;20:377–78.

[20] Škorpíková L, Vadlejch J, Ilgová J, Plhal R, Drimaj J, Mikulka O, et al. Molecular uncovering of important helminth species in wild ruminants in the Czech Republic. Front Vet Sci. 2025;12: 1544270. 39968104 10.3389/fvets.2025.1544270 PMC11832707

[21] Hofmann RR. Evolutionary steps of ecophysiological adaptation and diversification of ruminants: a comparative view of their digestive system. Oecologia. 1989;78:443–57. 28312172 10.1007/BF00378733

[22] Homolka M, Heroldová M. Native red deer and introduced chamois: foraging habits and competition in a subalpine meadow-spruce forest area. Folia Zool. 2001;50:89–98.

[23] Albery GF, Kenyon F, Morris A, Morris S, Nussey DH, Pemberton JM. Seasonality of helminth infection in wild red deer varies between individuals and between parasite taxa. Parasitology. 2018;145:1410–20. 29519265 10.1017/S0031182018000185 PMC6137381

[24] Chambers A, Candy P, Green P, Sauermann C, Leathwick D. Seasonal output of gastrointestinal nematode eggs and lungworm larvae in farmed Wapiti and red deer of new Zealand. Vet Parasitol. 2022;303: 109660. 35168114 10.1016/j.vetpar.2022.109660

[25] Reslová N, Škorpiková L, Kyriánová IA, Vadlejch J, Höglund J, Skuce P, et al. The identification and semi-quantitative assessment of Gastrointestinal nematodes in faecal samples using multiplex real-time PCR assays. Parasit Vectors. 2021;14:391. 34372893 10.1186/s13071-021-04882-4 PMC8351436

[26] Beck HE, Zimmermann NE, McVicar TR, Vergopolan N, Berg A, Wood EF. Present and future Köppen-Geiger climate classification maps at 1-km resolution. Sci Data. 2018;5: 180214. 30375988 10.1038/sdata.2018.214 PMC6207062

[27] Ahrens CD. Meteorology today: an introduction to weather, climate, and the environment. Belmont: Cengage Learning Canada Inc; 2015.

[28] Kotrlá B, Kotrlý A, Koždoň O. Studies on the specifity of the nematode *Ashworthius sidemi* schulz, 1933. Acta Vet Brno. 1976;45:123–26.

[29] Karbowiak G, Demiaszkiewicz AW, Pyziel AM, Wita I, Moskwa B, Werszko J, et al. The parasitic fauna of the European Bison (*Bison bonasus*) (Linnaeus, 1758) and their impact on the conservation. Part 2 the structure and changes over time. Acta Parasitol. 2014;59:372–9. 25119349 10.2478/s11686-014-0253-z

[30] Kołodziej-Sobocińska M, Demiaszkiewicz AW, Pyziel AM, Kowalczyk R. Increased parasitic load in captive-released European bison (*Bison bonasus*) has important implications for reintroduction programs. EcoHealth. 2018;15:467–71. 29549590 10.1007/s10393-018-1327-4 PMC6132417

[31] Vadlejch J, Kyriánová IA, Rylková K, Zikmund M, Langrová I. Health risks associated with wild animal translocation: a case of the European bison and an alien parasite. Biol Invasions. 2017;19:1121–5.

[32] Wyrobisz-Papiewska A, Kowal J, Nosal P, Chovancová G, Rehbein S. Host specificity and species diversity of the ostertagiinae Lopez-Neyra, 1947 in ruminants: a European perspective. Parasit Vectors. 2018;11:369. 29954435 10.1186/s13071-018-2958-6 PMC6022717

[33] Formenti N, Viganó R, Fraquelli C, Trogu T, Bonfanti M, Lanfranchi P, et al. Increased hormonal stress response of Apennine chamois induced by interspecific interactions and anthropogenic disturbance. Eur J Wildl Res. 2018;64: 68.

[34] Magdálek J, Škorpíková L, McFarland C, Vadlejch J. An alien parasite in a changing world – *Ashworthius sidemi* has lost its traditional seasonal dynamics. Front Vet Sci. 2023;10: 1279073. 38026660 10.3389/fvets.2023.1279073 PMC10646533

[35] Ovcharenko DA. Seasonal dynamics and development of *Ashworthius sidemi* (Trichostrongylidae), *Oesophagostomum radiatum* and *O. venulosum* (Strongylidae) of *Cervus Nippon hortulorum*. Parazitologiia. 1968;2:470–4.

[36] Drózdz J, Demiaszkiewicz AW, Lachowicz J. Expansion of the Asiatic parasite *Ashworthius sidemi* (Nematoda, Trichostrongylidae) in wild ruminants in Polish territory. Parasitol Res. 2003;89:94–7. 12489006 10.1007/s00436-002-0675-7

[37] Beaumelle C, Toïgo C, Papet R, Benabed S, Beurier M, Bordes L, et al. Cross-transmission of resistant gastrointestinal nematodes between wildlife and transhumant sheep. Peer Community J. 2024;4: 103.

[38] Beaumelle C, Redman E, Verheyden H, Jacquiet P, Bégoc N, Veyssière F, et al. Generalist nematodes dominate the nemabiome of roe deer in sympatry with sheep at a regional level. Int J Parasitol. 2022;52:751–61. 36183847 10.1016/j.ijpara.2022.07.005

[39] Rinaldi L, Catelan D, Musella V, Cecconi L, Hertzberg H, Torgerson PR, et al. *Haemonchus contortus*: spatial risk distribution for infection in sheep in Europe. Geospat Health. 2015;9:325–31. 25826314 10.4081/gh.2015.355

[40] Rose H, Caminade C, Bolajoko MB, Phelan P, van Dijk J, Baylis M, et al. Climate-driven changes to the spatio-temporal distribution of the parasitic nematode, *Haemonchus contortus*, in sheep in Europe. Glob Chang Biol. 2016;22:1271–85. 26482823 10.1111/gcb.13132

[41] Gibbs HC. Hypobiosis in parasitic Nematodes—An update. Adv Parasitol. 1986;25:129–74. 3535434

[42] Sargison ND, Wilson DJ, Bartley DJ, Penny CD, Jackson F. Haemonchosis and teladorsagiosis in a Scottish sheep flock putatively associated with the overwintering of hypobiotic fourth stage larvae. Vet Parasitol. 2007;147:326–31. 17531390 10.1016/j.vetpar.2007.04.011

[43] O’Connor LJ, Walkden-Brown SW, Kahn LP. Ecology of the free-living stages of major trichostrongylid parasites of sheep. Vet Parasitol. 2006;142:1–15. 17011129 10.1016/j.vetpar.2006.08.035

[44] Rose H, Wang T, van Dijk J, Morgan ER. Gloworm-fl: a simulation model of the effects of climate and climate change on the free-living stages of gastro-intestinal nematode parasites of ruminants. Ecol Modell. 2015;297:232–45.

[45] Wang T, Avramenko RW, Redman EM, Wit J, Gilleard JS, Colwell DD. High levels of third-stage larvae (L3) overwinter survival for multiple cattle gastrointestinal nematode species on Western Canadian pastures as revealed by ITS2 rDNA metabarcoding. Parasit Vectors. 2020;13: 458. 32912326 10.1186/s13071-020-04337-2 PMC7488095

[46] van Dijk J, David GP, Baird G, Morgan ER. Back to the future: developing hypotheses on the effects of climate change on ovine parasitic gastroenteritis from historical data. Vet Parasitol. 2008;158:73–84. 18824303 10.1016/j.vetpar.2008.08.006

[47] Corlatti L, Béthaz S, von Hardenberg A, Bassano B, Palme R, Lovari S. Hormones, parasites and male mating tactics in alpine chamois: identifying the mechanisms of life history trade-offs. Anim Behav. 2012;84:1061–70.

[48] Corlatti L, Lorenzetti C, Bassano B. Parasitism and alternative reproductive tactics in Northern chamois. Ecol Evol. 2019;9:8749–58. 31410277 10.1002/ece3.5427 PMC6686307

[49] McMahon C, Gordon AW, Edgar HWJ, Hanna REB, Brennan GP, Fairweather I. The effects of climate change on ovine parasitic gastroenteritis determined using veterinary surveillance and meteorological data for Northern Ireland over the period 1999–2009. Vet Parasitol. 2012;190:167–77. 22789298 10.1016/j.vetpar.2012.06.016

[50] Evans MJ, Corripio-Miyar Y, Hayward A, Kenyon F, McNeilly TN, Nussey DH. Antagonism between co-infecting gastrointestinal nematodes: a meta-analysis of experimental infections in sheep. Vet Parasitol. 2023;323: 110053. 37879240 10.1016/j.vetpar.2023.110053

[51] van Dijk J, Sargison ND, Kenyon F, Skuce PJ. Climate change and infectious disease: helminthological challenges to farmed ruminants in temperate regions. Animal. 2010;4:377–92. 22443942 10.1017/S1751731109990991

[52] Dobson RJ, Barnes EH, Birclijin SD, Gill JH. Researchnote the survival of *Ostertagia circumcincta* and *Trichostrongylus colubriformis* in faecal culture as a source of bias in apportioning egg counts to worm species. Int J Parasitol. 1992;22:1005–8. 1459776 10.1016/0020-7519(92)90060-x

[53] Mapes CJ, Coop RL. Effect of concureent and terminated infections of *Haemonchus contortus* on the development and reproductive capacity of *Nematodirus Battus*. J Comp Pathol. 1971;81:479–92. 5135638 10.1016/0021-9975(71)90075-2

