# Supplementary material for: Seasonal dynamics and potential interactions of haematophagous abomasal nematodes in two chamois populations in the Czech Republic
Source: BMC Vet Res. 2025 Sep 1;21:532. doi: 10.1186/s12917-025-04992-6 (PMC12400541; doi:10.1186/s12917-025-04992-6)
Supplement: Supplementary file 1 — Supplementary Material 1. [file 12917_2025_4992_MOESM1_ESM.docx]

| Response variable | Factor | Estimate±SD | Z-value | p-value |
| --- | --- | --- | --- | --- |
| Presence/absence of *H. contortus* | (Intercept) | 0.134±0.366 | 0.365 | 0.715 |
|  | Locality B | ≈ 0 | 0.000 | 1.000 |
|  | Spring | ≈ 0 | 0.000 | 1.000 |
|  | Summer | 0.714±0.541 | 1.319 | 0.187 |
|  | Winter | -0.980±0.541 | -1.813 | 0.069 |
|  | Spring*Locality B | -2.005±0.830 | -2.414 | **0.016** |
|  | Summer*Locality B | -0.714±0.749 | -0.953 | 0.340 |
|  | Winter*Locality B | -0.539±0.797 | -0.676 | 0.489 |
| Random effect | Variance | SD |  |  |
| Month:locality | 2.264e-09 | 4.759e-05 |  |  |

**Additional table 1**: Results of the generalized linear mixed model (GLMM) for probability of *Haemonchus contortus* DNA presence.

**Additional table 2**: Results of the generalized linear mixed model (GLMM) for relative amount of *Haemonchus contortus* DNA in positive samples.

| Response variable | Factor | Estimate±SD | Z-value | p-value |
| --- | --- | --- | --- | --- |
| Level of *H. contortus* DNA | (Intercept) | 12.42±0.680 | 18.256 | <0.001 |
|  | Spring | -3.408±0.905 | -3.766 | **<0.001** |
|  | Summer | 3.931±0.868 | 4.528 | **<0.001** |
|  | Winter | -2.617±0.947 | -2.762 | **0.006** |
|  | Locality B | -0.994±0.831 | -1.195 | 0.231 |
|  | Spring*Locality B | 8.083±1.417 | 5.705 | **<0.001** |
|  | Summer*Locality B | 0.372±1.108 | 0.336 | 0.737 |
|  | Winter*Locality B | -2.559±1.349 | -1.897 | 0.058 |
| Random effect | Variance | SD |  |  |
| Month:Locality | 0.257 | 0.507 |  |  |


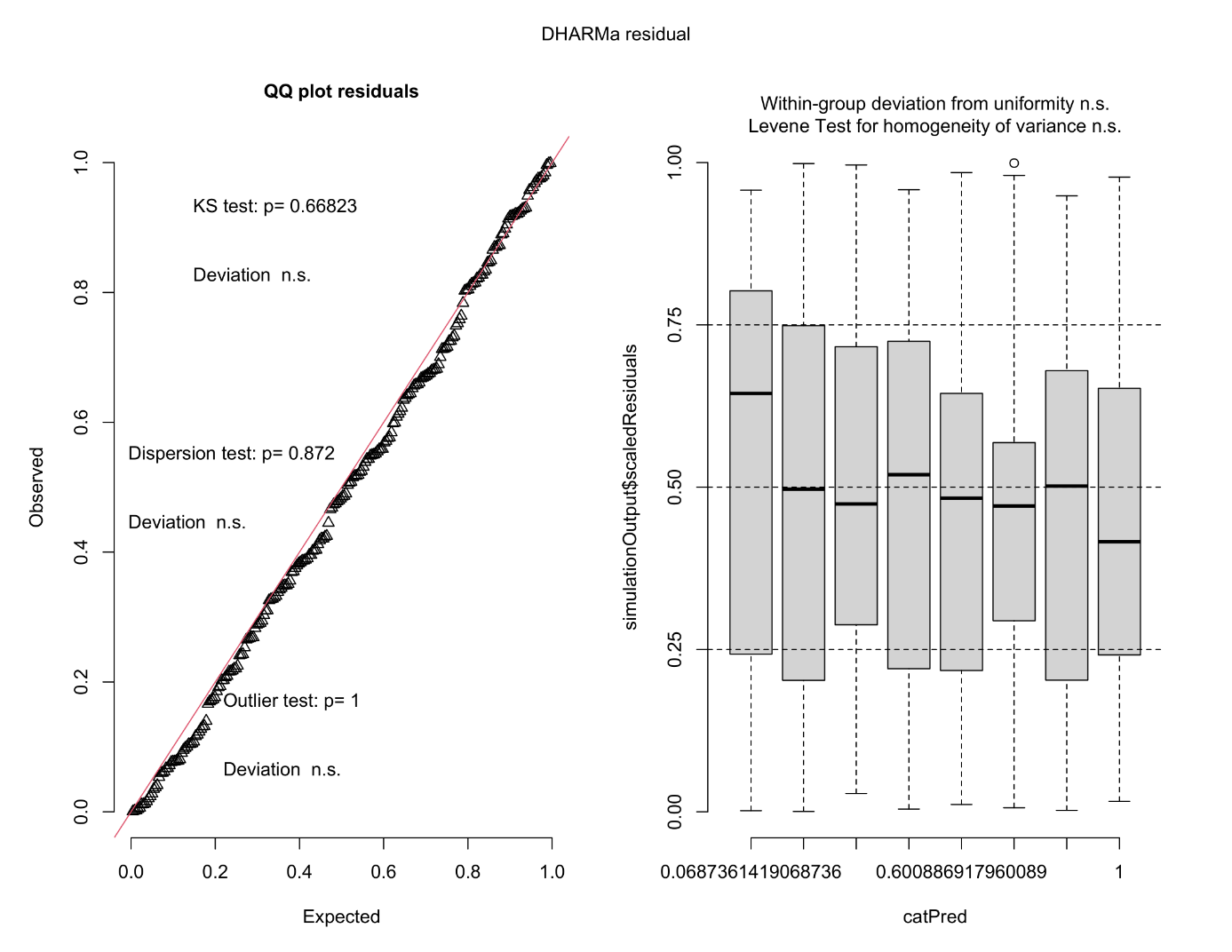


**Additional figure 1**: Simulated residuals plotted against fitted values to assess uniformity and detect potential patterns indicating model misspecification for Model 1 (Presence/absence of *H. contortus*).


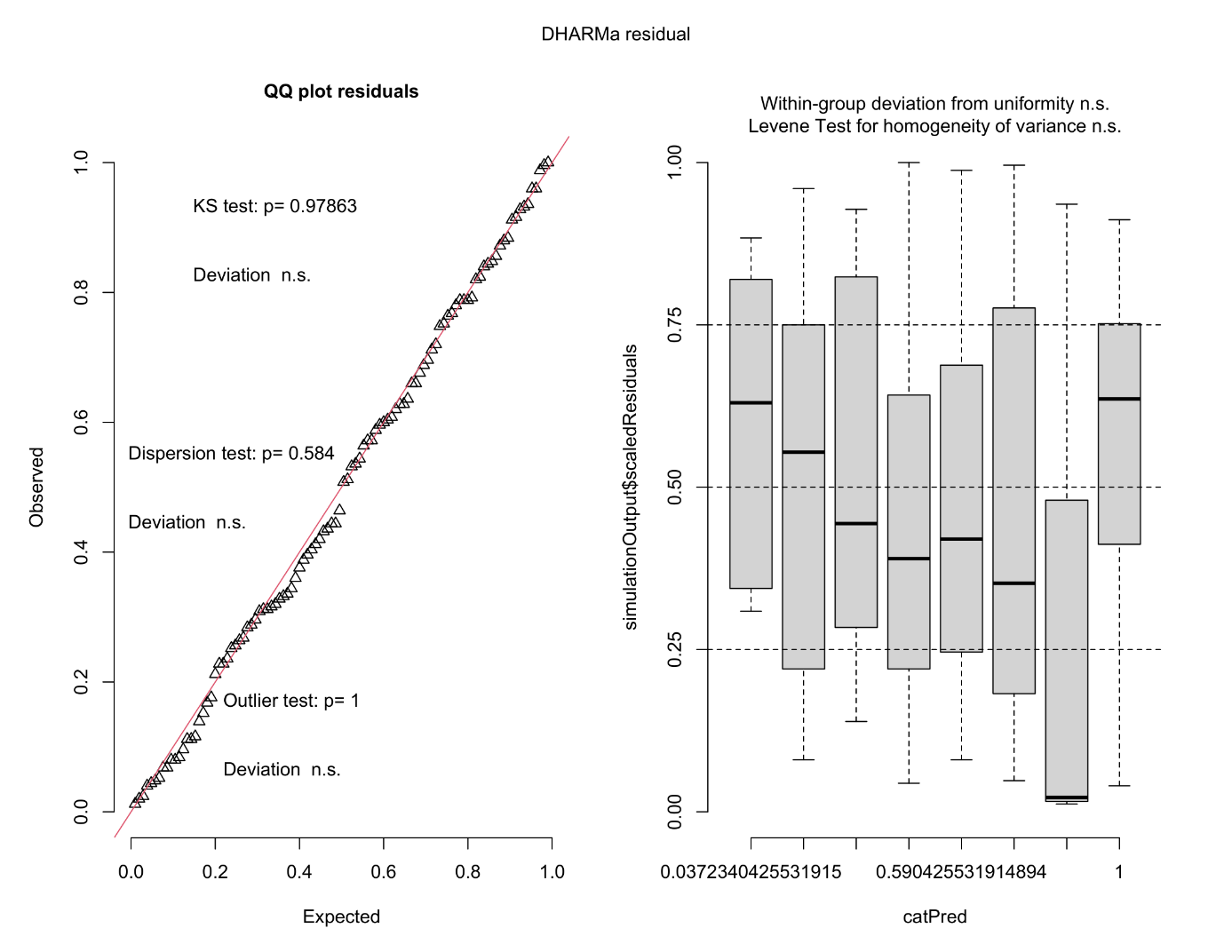


**Additional figure 2** Simulated residuals plotted against fitted values to assess uniformity and detect potential patterns indicating model misspecification for Model 2 (Level of *H. contortus* DNA).
